# The impact of the Lancet Commission definition of obesity on its prevalence and implications on long-term cardiovascular-kidney-metabolic outcomes in East Asians: Observational study of two community-based cohorts

**DOI:** 10.1371/journal.pmed.1004749

**Published:** 2026-02-09

**Authors:** David T. W. Lui, Carol H. Y. Fong, Xincheng Zou, Aimin Xu, Hung Fat Tse, Jean Woo, Tai Hing Lam, Yu Cho Woo, Bernard M. Y. Cheung, Edward Janus, Karen S. L. Lam, Kathryn C. B. Tan, Chi Ho Lee

**Affiliations:** 1 Department of Medicine, School of Clinical Medicine, Li Ka Shing Faculty of Medicine, University of Hong Kong, Hong Kong Special Administrative Region, People’s Republic of China; 2 State Key Laboratory of Pharmaceutical Biotechnology, Li Ka Shing Faculty of Medicine, University of Hong Kong, Hong Kong Special Administrative Region, People’s Republic of China; 3 Department of Medicine and Therapeutics, The Chinese University of Hong Kong, Hong Kong Special Administrative Region, People’s Republic of China; 4 The School of Public Health, Li Ka Shing Faculty of Medicine, University of Hong Kong, Hong Kong Special Administrative Region, People’s Republic of China; 5 Western Health Chronic Disease Alliance and Department of Medicine, Western Health-Melbourne Medical School, University of Melbourne, Parkville, Australia; Waymark, UNITED STATES OF AMERICA

## Abstract

**Background:**

The Lancet Commission proposed an update in January 2025 on the definition of obesity which requires at least one anthropometric measurement in addition to body mass index (BMI) to confirm excess adiposity. Also, the presence of obesity-related organ dysfunction is used to differentiate between clinical and pre-clinical obesity. We evaluated how applying the Lancet Commission proposed definition of obesity, which required an additional anthropometric measurement to verify excess adiposity, would affect its prevalence, and its implications on the cardiovascular-kidney-metabolic health.

**Methods and findings:**

We used two representative Chinese community-based cohorts and compared five categories of participants with (i) clinical obesity, (ii) preclinical obesity, (iii) BMI ≥25 kg/m^2^ without confirmed excess adiposity, (iv) overweight and (v) normal/underweight in the cross-sectional cohort for cardiometabolic risk profiles and in the longitudinal cohort for long-term cardiovascular-kidney-metabolic outcomes. In the cross-sectional cohort, the prevalence of obesity was 44.5% in men and 26.7% in women defined by the Asian BMI cutoff of ≥25.0 kg/m^2^, and decreased to 33.8% and 24.1%, respectively, using the Lancet Commission definition (BMI ≥ 25.0 kg/m^2^ and elevated waist circumference). Applying the Lancet Commission definition would reclassify a portion of individuals who are initially classified as having obesity based on BMI criteria alone (BMI ≥ 25.0 kg/m^2^) but with normal waist circumference to be non-obese (category iii). The individuals falling into category iii had an adverse cardiometabolic health profile which was intermediate among the five categories regarding insulin resistance and visceral adiposity (falling in between categories ii and iv). In the longitudinal cohort with a median follow-up of over 20 years, people with clinical obesity had the poorest cardiovascular-kidney-metabolic outcomes including all-cause mortality, whereas those reclassified as non-obese had an intermediate risk of adverse cardiovascular-kidney-metabolic outcomes among the five categories. The main limitation of the study was that all participants were Chinese and findings might not apply to other ethnic groups.

**Conclusion:**

Adoption of the Lancet Commission definition would classify a small proportion of individuals with BMI of ≥25.0 kg/m^2^ as non-obese. People with clinical obesity identified by the revised criteria had the highest risks of cardiovascular-kidney-metabolic outcomes including all-cause mortality, whereas individuals reclassified as non-obese had intermediate risks of cardiovascular-kidney-metabolic outcomes between those in pre-clinical obesity and overweight categories.

## Author summary

**Why was this study done?:**

- The Lancet Commission proposed an update in January 2025 on the definition of obesity which requires at least one anthropometric measurement in addition to body mass index (BMI) to confirm excess adiposity.
- Only two population-based cross-sectional studies, one on a United States cohort and one in the United Arab Emirates, have evaluated the impact of applying the Lancet Commission definitions on the prevalence of obesity, but these did not report long-term outcomes.

**What did the researchers do and find?:**

- This study used two representative Hong Kong Chinese community-based cohorts to analyse (i) the cardiometabolic risk profile of the participants with clinical/pre-clinical obesity and those who would be reclassified as non-obese based on the Lancet Commission definition cross-sectionally, and (ii) the long-term cardiovascular-kidney-metabolic outcomes over a median follow-up of over 20 years
- Application of the Lancet Commission definition led to reclassification into non-obese for 6.7% of the cohort who would otherwise have been diagnosed to have obesity based on BMI cut-off alone; these people were in the intermediate range of the spectrum of BMI and adiposity
- Clinical obesity presents the highest risk of adverse cardiovascular-kidney-metabolic outcomes, while people reclassified as non-obese still had more adverse long-term health impacts than those with normal body weight

**What do these findings mean?:**

- Compared to BMI-alone criteria, the Lancet Commission definition identifies people with clinical obesity to maximise benefits of timely intervention, but people who were diagnosed to have obesity based on BMI cut-off alone but reclassified as non-obese under the Lancet Commission definition still had higher risk of adverse outcomes than those with normal BMI.
- For public health, BMI is still an important index to raise awareness to prevent obesity.
- The main limitations of the study were that all participants were Chinese and the limited consideration of social and environmental determinants in relation to the various cardiovascular-kidney-metabolic outcomes.

## Abstract 3

In an observational study, David Lui and colleagues investigate the effect of a new obesity definition on the classification of individuals as overweight in two Chinese cohorts.

## Introduction

Body mass index (BMI) is calculated as body weight (kg) divided by the square of height (m^2^) [1]. BMI is commonly used to diagnose obesity in view of its convenience. However, there are certain limitations in using BMI to diagnose obesity. BMI does not adequately capture the body fat distribution [2]. It also does not inform practitioners of the heterogeneity of obesity and its outcomes in specific individuals or populations [3]. A blanket definition of obesity using a single BMI cutoff may lead to misclassification and result in both overdiagnosis or underdiagnosis of obesity status. In January 2025, the Lancet Commission proposed an update in the definition of obesity which requires an additional anthropometric measurement to reflect excess adiposity (thereafter referred to as the Lancet Commission definition) [4]. The consensus statement recommends excess or abnormal adiposity be confirmed by one of the following: (i) direct body fat measurement by dual energy X-ray absorptiometry (DXA) or bioimpedance; or (ii) at least one anthropometric criterion in addition to BMI; or (iii) at least two anthropometric criteria regardless of BMI. The anthropometric measurements could be waist circumference (WC), waist-to-hip ratio or waist-to-height ratio, using validated age, sex, and ethnicity-appropriate cut-off points. People with obesity under these new criteria are further classified into clinical and pre-clinical obesity according to the presence of organ dysfunction related to obesity: those who do not have obesity-related organ dysfunction are classified as having pre-clinical obesity, while those who have obesity-related organ dysfunction are classified as having clinical obesity. The implication of defining clinical and pre-clinical obesity is that people with these conditions should be regularly monitored and screened for obesity-related conditions including type 2 diabetes [4].

In current clinical practice, people are largely classified by BMI alone into different body weight categories. The BMI cut-off levels adopted in Asians include (i) <18.5 kg/m^2^ for underweight, (ii) 18.5–22.9 kg/m^2^ indicating normal, (iii) 23.0–24.9 kg/m^2^ defining overweight and (iv) ≥25.0 kg/m^2^ defining obesity, as recommended by the World Health Organisation, International Association for the Study of Obesity and International Obesity Task Force in 2000 [5]. These Asians BMI cut-offs were shown to be appropriate for Chinese in validation studies based on body fat determination by DXA [6] and mortality outcomes [7]. The introduction of the Lancet Commission definition of obesity will likely change the prevalence of obesity. The Lancet Commission definition mainly concerns people who have been classified as having obesity based on BMI alone, because some of these people could be reclassified into non-obese if their WC or body fat percentage do not reach the respective thresholds. It remains to be elucidated the extent of such a change, and the cardiometabolic risk profile of the people who are reclassified as non-obese according to the new definition. For Asians, the BMI cut-off for obesity is 25.0 kg/m^2^, while that for WC is ≥90.0 cm for men and ≥80.0 cm for women [8]. Whether including the WC criterion would result in the change in prevalence has not been studied. More importantly, the long-term outcomes of these people who are reclassified as non-obese have not been reported.

Hence, we examined the change in the prevalence of obesity using BMI alone and the Lancet Commission definition, as well as the cardiometabolic risk profile of the people who are reclassified as non-obese according to the Lancet Commission definition, using cross-sectional data from a contemporary community-based cohort (recruitment in 2019–2025). We further studied their long-term cardiovascular-kidney-metabolic outcomes using another community-based cohort of similar design (recruitment in 1994–1996) with a median follow-up of over 20 years.

## Methods

This study is reported as per the Strengthening the Reporting of Observational Studies in Epidemiology (STROBE) guideline (S1 STROBE Checklist). The current study represents the post-hoc analyses of two community-based cohorts prospectively built to track the evolution of the prevalence of cardiovascular diseases and risk factors over two decades. The analyses involved in this study were planned following the publication of the Lancet Commission definition in January 2025 to retrospectively study the impact of applying this set of definition on these two cohorts—the recruitment of which had already been complete.

### Study participants

Our study consisted of two parts, using two community-based cohorts with a similar study design—the Hong Kong Cardiovascular Risk Factor Prevalence Study (CRISPS) and the New Hong Kong Cardiovascular Risk Factor Prevalence Study (N-CRISPS). Details of the profile of the two cohorts have been described previously [9]. Briefly, CRISPS commenced in 1995 while N-CRISPS commenced in 2019. In both cohorts, 10 age–sex specific groups (25–34, 35–44, 45–54, 55–64, 65–74 years old) were recruited according to the World Health Organization (WHO) Monitoring Trends and Determinants in Cardiovascular Disease recommendation. At least 200 participants were included in each of the 10 age–sex specific groups to form representative samples of the Hong Kong Chinese population. The recruitment for CRISPS was done through randomly generated telephone numbers providing a random sample of the population, while the recruitment for N-CRISPS was done through mail invitations to residents of randomly selected households with addresses registered at the Census and Statistics Department. Fasting blood samples were collected and anthropometric parameters, including body height and weight, waist and hip circumferences, and blood pressure were measured using standardised procedures [10]. In CRISPS cohort, for practical purposes, WC was measured halfway between the xiphisternum and the umbilicus [11], which highly correlated with WHO recommendation of WC measurement halfway between the lower rib margin and iliac crest [12]. The correlation coefficient (*r*) was 0.981 (*p* < 0.001) demonstrated in a sub-cohort of CRISPS (*n* = 451), with an excellent agreement in the classification of central obesity (kappa coefficient = 0.865, *p* < 0.001). In N-CRISPS, WC was measured following WHO recommendation. Detailed questionnaires regarding lifestyle and health issues (including medical comorbidities) were administered by trained interviewers. Physical examination, and comprehensive metabolic assessments for hypertension (defined by blood pressure ≥140/90 mmHg or on anti-hypertensive medications), diabetes and dyslipidaemia (defined as fasting triglycerides ≥1.7 mmol/L, high density lipoprotein-cholesterol <1.3mmol/L in women and <1.0mmol/L in men, low density lipoprotein-cholesterol ≥3.4 mmol/L, or on lipid-lowering medications) were done, including a 75-g oral glucose tolerance test. The cohorts are linked to the electronic health records of the Hong Kong Hospital Authority which maintains an excellent record of patients’ diagnoses, laboratory investigations, prescriptions, and procedures, allowing satisfactory capture of clinical outcomes of the cohort participants.

Participants of CRISPS were invited to attend subsequent visits every 4–5 years (CRISPS-2 to -5; the last visit in CRISPS-5 was in 2016–2018) with similar assessment protocols including 75-g oral glucose tolerance tests. Thus, this longitudinal cohort provides detailed assessments of the evolution of glycaemic and other cardiovascular-kidney-metabolic status over two decades.

In N-CRISPS, assessment of liver, visceral, and subcutaneous fat was done in addition to the CRISPS protocol. Body fat was analysed using the bioelectrical impedance Tanita Body Composition Analyzer DC-430MA (Tanita Corp., Tokyo, Japan). Hepatic steatosis and fibrosis was determined based on controlled attenuation parameter (CAP) and liver stiffness (LS), respectively, using vibration-controlled transient elastography (VCTE) [13]. Furthermore, a sub-cohort of participants aged 55–74 years who agreed to return for a second visit and underwent whole-body DXA (Hologic Discovery A) for body composition were included in the present study for analysis.

### Definition of clinical and pre-clinical obesity according to the Lancet Commission

For the original definition of obesity defined by BMI alone, the Asian cutoff of ≥25.0 kg/m^2^ was adopted [8]. As for the Lancet Commission definition of obesity, we chose the combination of BMI ≥25.0 kg/m^2^ and elevated WC (≥90 cm in men; ≥80 cm in women) as these measurements can be practically applied in routine clinical practice with ethnically validated cut-off points [14]. In addition, body fat of ≥25% in men and ≥35% in women was considered to indicate excess adiposity [15]. According to the Lancet Commission, individuals with obesity are further classified into pre-clinical obesity (in which the individual does not have any obesity-related organ dysfunction) and clinical obesity (in which the individual has obesity-related organ dysfunction). The operational definitions for the obesity-related organ dysfunction are shown in S1 Table.

### Cross-sectional analysis using N-CRISPS

We used N-CRISPS to analyse the impact of the Lancet Commission definition of obesity on the prevalence of obesity compared to that when defined by BMI criteria alone. As mentioned above, the Lancet Commission definition of obesity mainly leads to changes in classification concerning the group of people classified as having obesity based solely on BMI criterion (≥25.0 kg/m^2^). Hence, in the current study, when the Lancet Commission definition of obesity was superimposed onto the BMI criteria, the participants were divided into five body weight categories [(i) clinical obesity, (ii) pre-clinical obesity, (iii) people with BMI ≥25 kg/m^2^ without confirmed excess adiposity (i.e., normal WC), (iv) overweight (BMI 23.0–24.9 kg/m^2^), and (v) normal/underweight (BMI <23.0 kg/m^2^)]. We examined the cardiometabolic risk profile of each category. Stratifications were done according to sex and age (<55 and ≥55 years).

Sensitivity analysis was done by additionally including percentage of body fat measured by bioelectric impedance for confirmation of excess adiposity, so that BMI ≥25 kg/m^2^ with elevated WC or increased percentage of body fat would define obesity.

### Longitudinal analysis using CRISPS

We used CRISPS cohort to study the long-term impact of using the Lancet Commission definition of obesity on the incidence of adverse cardiovascular-kidney-metabolic outcomes (cardiovascular diseases, kidney outcomes, cancer, diabetes and all-cause mortality) upon median follow-up of over 20 years. The cohort was divided into five categories as described above according to baseline status, and was followed up from the index date (the date of recruitment into CRISPS) to occurrence of outcomes of interest, death, or 31 December 2024, whichever was earlier. Participants meeting those outcome definitions before the baseline and those who had less than one year of follow-up were excluded.

Incident cardiovascular diseases were defined by ICD‐9 codes 402, 404, 410‐414, 425‐447, 518.4, which included acute myocardial infarction, angina, stroke, and heart failure [16]. All these events were verified from the Hospital Authority database or private practitioners, including the dates of the events and the discharge diagnosis. Incident kidney outcomes were defined as a composite of eGFR <15mL/min, dialysis and kidney transplantation [17]. Incident diabetes was defined by fasting glucose ≥7.0 mmol/L, HbA1c ≥6.5%, 2-hour glucose >11.0mmol/L, initiation of anti-diabetic medications, or physician-diagnosed diabetes. Incident cancer was defined by ICD-9 CM codes 140-165,170-176,179-209 and 230-239 [18]. For participants who died, causes and dates of death were available from the Hong Kong Death Registry. Hence, all-cause mortality could be analysed with reasonable accuracy.

### Statistical analyses

All statistical analyses were done using SPSS version 28 and R (version 4.3.2). Sankey diagrams were generated using the **ggplot2** and **ggsankey** packages, while the **survival** and **rms** packages were used for regression analysis. Descriptive statistics were presented as mean (standard deviation, SD) for data with normal distribution, median (interquartile range) for non-normal data, or number with percentage as appropriate. Variables with non-normal distributions were log-transformed prior to analysis. In the cross-sectional cohort, between-group comparisons were done using ANOVA for continuous variables and either Chi-squared or Fisher’s exact test for categorical variables. The Cochran–Armitage trend test was applied to assess binary outcomes across ordered groups, while *F* tests were used to evaluate linear trends in continuous outcomes. To control the family-wise error rate in multiple testing, the Holm–Bonferroni approach, which uses a stepwise adjustment procedure, was applied. In the longitudinal cohort, survival analysis techniques were used to compare the long-term risk of different cardiovascular-kidney-metabolic outcomes across the five categories. In the survival analyses, we excluded individuals with a prior history of a given condition when we evaluated the incidence of that condition. Kaplan–Meier product-limit estimates were used to summarise survival over the follow-up period for each category. Since the proportional hazard assumption assessed via the Schoenfeld Residuals Test in Cox regression was violated, the Accelerated Failure Time (AFT) model was used to assess the risk of long-term outcomes across the five categories. All models were adjusted for sex, age, smoking status, current alcohol consumption, exercise, and educational attainment. Model performance was assessed using the Akaike Information Criterion (AIC), where a lower AIC indicated a better fit. Acceleration Factors (AF) were presented as estimates for each category, interpreted as the following: AF > 1 indicated that the exposure (the respective body weight category) improved survival time; AF < 1 indicated that the exposure reduced survival time; and AF = 1 indicated no effect of the exposure on survival time. Missing data in CRISPS and N-CRISPS were handled using complete-case analyses.

In the longitudinal analysis of CRISPS cohort, to account for possibility of out-migration of the CRISPS participants, we performed a sensitivity analysis of the association between body weight categories and cardiovascular-kidney-metabolic outcomes after excluding individuals who did not have any follow-up or clinical assessments in the public hospitals operated by the Hospital Authority (Hong Kong Hospital Authority provides comprehensive healthcare services to over 90% of the Hong Kong population).

### Ethics statement

Ethics approval has been obtained from the Institutional Review Board of the University of Hong Kong/Hospital Authority, Hong Kong West Cluster (approval numbers: EC-849-96; UW 05-296 T/959; UW 08-297; UW 16-048; UW 18–610). Written consent has been provided by all participants.

## Results

### Cross-sectional analysis of prevalence of obesity: results from N-CRISPS

Table 1 summarises the characteristics of all 3,258 participants in N-CRISPS (mean BMI: 24.9 kg/m^2^ [SD 3.7] in men; 23.2 kg/m^2^ [SD 3.9] in women). The prevalence of obesity defined by BMI alone was 36% overall (44.5% in men; 26.7% in women). Hypertension, diabetes and dyslipidaemia were common, accounting for 30%, 12% and 56% respectively.

**Table 1:** Clinical characteristics of N-CRISPS participants (*N* = 3,258).

	Total	Men	Women
Number	3,258	1,635 (50%)	1,623 (50%)
Age, years	49 (SD: 14)	49 (SD: 14)	49 (SD: 14)
25–34	733 (22.5%)	347 (21.2%)	386 (23.8%)
35–44	690 (21.2%)	381 (23.3%)	309 (19.0%)
45–54	600 (18.4%)	277 (16.9%)	323 (19.9%)
55–64	602 (18.5%)	310 (19.0%)	292 (18.0%)
65–74	633 (19.4%)	320 (19.6%)	313 (19.3%)
Smoking status			
Never	2,503 (76.8%)	1,052 (64.3%)	1,451 (89.4)
Former	409 (12.6%)	312 (19.1%)	97 (6.0%)
Current	346 (10.6%)	271 (16.6%)	75 (4.6%)
Alcohol consumption			
Never	957 (29.4%)	333 (20.4%)	624 (38.4%)
Social (<1 day per month)	1,570 (48.2%)	819 (50.1%)	751 (46.3%)
Current (at least 1–3 days per month)	346 (10.6%)	371 (16.6%)	75 (4.6%)
Regular exercise	1814 (55.7%)	974 (59.6%)	840 (51.8%)
Educational attainment			
No schooling/primary	237 (7.3%)	91 (5.6%)	146 (9.0%)
Secondary	1,092 (33.5%)	498 (30.5%)	594 (36.6%)
Tertiary or above	1929 (59.2%)	1,046 (64.0%)	883 (54.4%)
**Anthropometric parameters**			
BMI, kg/m^2^	24.1 (SD: 3.9)	24.9 (SD: 3.7)	23.2 (SD: 3.9)
<18.5	133 (4.1%)	32 (2.0%)	101 (6.2%)
18.5–22.9	1,276 (39.2%)	482 (29.5%)	794 (48.9%)
23.0–24.9	689 (21.1%)	394 (24.1%)	295 (18.2%)
25.0–29.9	929 (28.5%)	588 (36.0%)	341 (21.0%)
≥30.0	231 (7.1%)	139 (8.5%)	92 (5.7%)
Waist circumference^*^, cm	82 (SD: 11)	88 (SD: 10)	77 (SD: 10)
Elevated waist circumference^*^ *(≥90 cm for men; ≥80 cm for women)*	1,203 (36.9%)	626 (38.3%)	577 (35.6%)
**Comorbidities**			
Hypertension	977 (30.0%)	613 (37.5%)	364 (22.4%)
Glycaemic status			
NGT	2,253 (69.2%)	1,104 (67.5%)	1,149 (70.8%)
Prediabetes	615 (18.9%)	318 (19.4%)	297 (18.3%)
Diabetes	390 (11.9%)	213 (13.1%)	177 (10.9%)
Dyslipidaemia	1824 (56.0%)	1,029 (62.9%)	795 (49.0%)
The cluster of hyperglycaemia, hypertriglyceridaemia and low HDL-C	169 (5.2%)	74 (4.5%)	95 (5.9%)
History of cardiovascular disease	157 (4.8%)	94 (5.7%)	63 (3.9%)
History of cancer	103 (3.2%)	29 (1.8%)	74 (4.6%)
CKD (eGFR <60 mL/min/1.73m^2^)	53 (1.6%)	33 (2%)	20 (1.2%)
NAFLD	1,249 (39%)	757 (47%)	492 (31%)

Data were presented as mean (standard deviation, SD) or number (%).

*N = 3257.

Abbreviations: BMI, body mass index; NGT, normal glucose tolerance; HDL-C, high density lipoprotein cholesterol; CKD, chronic kidney disease; eGFR, estimated glomerular filtration rate; NAFLD, non-alcoholic fatty liver disease.

Upon application of the Lancet Commission definition (defined by both elevated BMI and WC), the overall prevalence of obesity decreased, with a greater reduction in men (from 44.5% to 33.8%) than in women (26.7% to 24.1%), and more so in the younger age groups (Table 2). The prevalence of clinical obesity was higher in men than in women, and in the older age groups.

**Table 2:** Prevalence of obesity in N-CRISPS cohort based on different criteria of obesity.

	Number	Obesity defined by BMI 25 kg/m^2^ alone	Obesity defined by elevated BMI and confirmed excess adiposity (elevated waist circumference)		
			**Preclinical Obesity**	**Clinical Obesity**	**Total**
**Men**					
25–54y	1,005	457 (45.5%)	120 (11.9%)	199 (19.8%)	319 (31.7)
55–74y	630	270 (42.9%)	35 (5.6%)	198 (31.4%)	233 (37.0)
***Total***	1,635	727 (44.5%)	155 (9.5%)	397 (24.3%)	552 (33.8)
**Women**					
25–54y	1,018	248 (24.4%)	110 (10.8%)	101 (9.9%)	211 (20.7)
55–74y	605	185 (30.6%)	57 (9.4%)	123 (20.3%)	180 (29.8)
***Total***	1,623	433 (26.7%)	167 (10.3%)	224 (13.8%)	391 (24.1)

BMI, body mass index.

The group who was previously classified to have obesity based on BMI alone, but reclassified as non-obese based on the Lancet Commission definition is labelled as ‘BMI ≥25kg/m^2^ without confirmed excess adiposity’ in the present study. S1 Fig shows the stratification of the N-CRISPS cohort into the five categories. The proportion of people reclassified as non-obese in the Lancet Commission definition was greater in men (14% in the younger subgroup and 6% in the older subgroup) than in women (only 4% in younger subgroup and 1% in the older subgroup). To place it in the context of people with BMI ≥25 kg/m^2^ (727 men and 433 women) (S2 Fig), reclassification to non-obese occurred in 30% in the younger men (<55 years old), 14% in the older men (≥55 years old), 15% in the younger women and 3% in the older women.

We further compared the cardiometabolic risk profile across the five categories (Table 3). People who had BMI ≥25 kg/m^2^ without confirmed excess adiposity had a cardiometabolic risk profile that was intermediate between clinical/pre-clinical obesity and BMI <23 kg/m^2^: best exemplified by the degree of insulin resistance indicated by HOMA-IR and the presence of hepatic steatosis indicated by CAP. There was a graded decrease in the degree of insulin resistance and liver fat from clinical obesity, pre-clinical obesity, BMI ≥25.0 kg/m^2^ without confirmed excess adiposity, overweight to normal/underweight categories (both *p* for trend <0.001). Direct measurements of adiposity via bioelectric impedance and whole-body DXA showed similar trends (*p* for trend <0.001) as CAP and HOMA-IR. In the bioelectrical impedance analysis, the group with BMI ≥25 kg/m^2^ without confirmed excess adiposity showed percentage fat mass and visceral fat rating which were intermediate between clinical/pre-clinical obesity and those with BMI <25 kg/m^2^ (Table 3). These observations were further shown in the subgroup with available whole-body DXA measurements—both fat mass/height^2^ and amount of visceral adipose tissue were also intermediate (S2 Table). There was a graded increase in glycaemia and blood pressure readings across the five categories from normal/underweight to clinical obesity (*p* for trend <0.01), although the number of people with coronary artery disease and stroke were relatively few to allow further comparisons. While there was a graded increase in the proportion of dyslipidaemia across the five categories (*p* for trend <0.001), it was mainly driven by the lower HDL-cholesterol and higher triglyceride levels due to insulin resistance, whereas LDL-cholesterol remained more or less similar across the five categories.

**Table 3:** Cardiometabolic risk profiles of men and women in the N-CRISPS cohort, classified according to the Lancet Commission definition of obesity.

	Non-obese group			Obesity group		*P* for trend
	Normal/ underweight BMI <23 kg/m^2^	Overweight BMI 23–24.9 kg/m^2^	BMI ≥25 kg/m^2^ without confirmed excess adiposity *(Reference group)*	Preclinical Obesity	Clinical Obesity	
**Men (*N* = 1,635)**						
**Number**	514	393	176	155	397	**–**
Age, years	48.3 (SD: 14.9)*	49.5 (SD: 14.3)*	43.5 (SD: 12.5)	44.9 (SD: 11.9)	53.4 (SD: 12.7)*	**0.002**
Prediabetes	67 (13.0%)	77 (19.6%)	24 (13.6%)	29 (18.7%)	121 (30.5%)*	<**0.001**
Diabetes	36 (7.0%)	45 (11.4%)	16 (9.1%)	7 (4.5%)	109 (27.5%)*	<**0.001**
Hypertension	107 (20.8%)*	139 (35.4%)	55 (31.3%)	0 (0%)*	312 (78.6%)*	<**0.001**
Dyslipidaemia	262 (51.0%)*	241 (61.3%)	120 (68.2%)	97 (62.6%)	309 (77.8%)	<**0.001**
Coronary artery disease	17 (3.3%)	20 (5.1%)	7 (4.0%)	2 (1.3%)	22 (5.5%)	0.349
Stroke	7 (1.4%)	12 (3.1%)	1 (0.6%)	0 (0%)	10 (2.5%)	0.739
eGFR (mL/min/1.73m^2^)	94.7 (SD: 15.0)	91.5 (SD: 15.6)*	95.6 (SD: 14.3)	95.6 (SD: 13.2)	89.5 (SD: 16.9)*	<**0.001**
**People not known to have diabetes (*N* = 1,505)**	492	363	166	154	330	–
Undiagnosed diabetes	14 (2.8%)	15 (4.1%)	6 (3.6%)	6 (3.9%)	42 (12.7%)*	**<0.001**
FG, mmol/L	4.9 (SD: 0.6)	5.0 (SD: 0.9)	4.9 (SD: 0.4)	5.0 (SD: 0.5)	5.3 (SD: 0.8)*	**<0.001**
2hrG, mmol/L	5.9 (SD: 2.2)	6.4 (SD: 2.6)	6.0 (SD: 2.0)	6.6 (SD: 2.1)	7.8 (SD: 3.0)*	**<0.001**
HbA1c, %	5.4 (SD: 0.4)	5.5 (SD: 0.6)	5.4 (SD: 0.4)	5.5 (SD: 0.4)	5.7 (SD: 0.6)*	**<0.001**
HOMA-IR^ϯ^	1.2 (0.9-1.7)*	1.6 (1.3-2.2)*	1.8 (14-2.4)	2.3 (1.8-3.1)*	3.1 (2.1-4.6)*	**<0.001**
**People not on anti-hypertensive medications (*N* = 1,318)**	462	323	152	155	226	**–**
SBP, mmHg	123 (SD: 13)*	126 (SD: 14)	128 (SD: 16)	124 (SD: 8.3)*	137 (SD: 15)*	**<0.001**
DBP, mmHg	78 (SD: 8.4)*	81 (SD: 9.5)*	82 (SD: 10)	81 (SD: 5)*	90 (SD: 10)*	**<0.001**
**People not on lipid lowering medications (*N* = 1,354)**	452	322	157	145	278	**–**
HDL-C, mmol/L	1.60 (SD: 0.41)*	1.44 (SD: 0.32)	1.39 (SD: 0.31)	1.31 (SD: 0.27)*	1.24 (SD: 0.30)*	**<0.001**
LDL-C, mmol/L	3.1 (SD: 0.9)*	3.2 (SD: 0.8)	3.4 (SD: 0.9)	3.3 (SD: 0.8)	3.3 (SD: 0.8)*	**0.001**
TG, mmol/L^ϯ^	0.9 (0.7-1.3)*	1.1 (0.8-1.6)*	1.2 (0.8-1.7)	1.2 (0.9-1.8)	1.4 (1.0-2.2)*	**<0.001**
**People without hepatitis B/C, or drugs associated with fatty liver (*N* = 1,512)**	481	364	163	142	362	**–**
VCTE						
CAP, dB/m	218 (SD: 31)*	243 (SD: 36)	246 (SD: 39)	280 (SD: 42)*	298 (SD: 41)*	**<0.001**
LS, kPa^ϯ^	4.5 (3.9-5.1)*	4.6 (4.0-5.2)	4.7 (4.1-5.2)	4.7 (4.3-5.4)*	5.7 (4.8-6.7)*	**<0.001**
**Bioelectric impedance analysis**						
%Fat	17.3 (SD: 4.1)*	21.7 (SD: 2.5)*	23.6 (SD: 2.2)	26.7 (SD: 3.0)*	28.0 (SD: 3.6)*	**<0.001**
Visceral Fat Rating	8.1 (SD: 3.5)*	11.8 (SD: 2.4)*	12.6 (SD: 1.9)	14.3 (SD: 2.1)*	16.2 (SD: 2.3)*	**<0.001**
**Women (*N* = 1,623)**						
**Numbe**r	895	295	42	167	224	
Age, years	46.5 (SD: 14.5)*	51.3 (SD: 13.9)*	41.0 (SD: 11.3)	48.8 (SD: 12.9)*	55.1 (SD: 12.7)*	<**0.001**
Prediabetes	116 (13.0%)	68 (23.1%)	7 (16.7%)	42 (25.1%)	64 (28.6%)	**<0.001**
Diabetes	58 (6.5%)	27 (9.2%)	1 (2.4%)	13 (7.8%)	78 (34.9%)*	**<0.001**
Hypertension	125 (14.0%)	72 (24.4%)	8 (19.0%)	0 (0%)*	159 (71.0%)*	**<0.001**
Dyslipidaemia	347 (38.8%)	145 (49.2%)	22 (52.4%)	95 (56.9%)	186 (83.0%)*	**<0.001**
Coronary artery disease	16 (1.8%)	8 (2.7%)	1 (2.4%)	1 (0.6%)	18 (8.0%)	**<0.001**
Stroke	13 (1.5%)	7 (2.4%)	1 (2.4%)	0 (0%)	7 (3.1%)	0.059
eGFR (mL/min/1.73m^2^)	100 (SD: 16.2)	96.6 (SD: 15.0)*	105 (SD:13.1)	98.7 (SD: 14.8)	93.7 (SD: 15.7)*	<**0.001**
**People not known to have diabetes (*N* = 1,540)**	865	283	42	164	186	
Undiagnosed diabetes	28 (3.2%)	15 (5.3%)	1 (2.4%)	10 (6.1%)	40 (21.5%)*	**<0.001**
FG, mmol/L	4.7 (SD: 0.5)	5.0 (SD: 0.7)*	4.8 (SD: 0.6)	5.0 (SD: 0.5)	5.4 (SD: 1.0)*	**<0.001**
2hrG, mmol/L	6.1 (SD: 2.0)	6.9 (SD: 2.5)*	6.1 (SD: 1.9)	7.0 (SD: 2.1)*	8.9 (SD: 3.3)*	**<0.001**
HbA1c, %	5.3 (SD: 0.4)	5.5 (SD: 0.5)*	5.3 (SD: 0.3)	5.5 (SD: 0.4)*	5.8 (SD: 0.6)*	**<0.001**
HOMA-IR^ϯ^	1.3 (0.9-1.7)*	1.7 (1.3-2.5)	1.8 (1.5-2.3)	2.3 (1.7-3.0)*	2.9 (2.0-4.7)*	**<0.001**
**People not on anti-hypertensive medications (*N* = 1,397)**	826	252	39	167	113	**–**
SBP, mmHg	112 (SD: 15)	118 (SD: 15)	116 (SD: 14)	118 (SD: 11)	131 (SD: 16)*	**<0.001**
DBP, mmHg	72 (SD: 9.2)*	76 (SD: 8.7)	76 (SD: 9.5)	76 (SD: 7.5)	85 (SD: 9.4)*	**<0.001**
**People not on lipid lowering medications (*N* = 1,420)**	803	262	39	153	163	**–**
HDL-C, mmol/L	1.90 (SD: 0.42)*	1.68 (SD: 0.43)	1.73 (SD: 0.44)	1.55 (SD: 0.35)*	1.40 (SD: 0.32)*	**<0.001**
LDL-C, mmol/L	2.9 (SD: 0.8)	3.0 (SD: 0.9)	2.9 (SD: 0.7)	3.1 (SD: 0.8)	3.2 (SD: 0.9)	**<0.001**
TG, mmol/L^ϯ^	0.8 (0.6-1.0)	1.0 (0.7-1.4)	0.8 (0.6-1.0)	1.0 (0.8-1.5)*	1.5 (1.0-2.0)*	**<0.001**
**People without hepatitis B/C, or drugs associated with fatty liver (*N* = 1,498)**	829	273	35	158	203	**–**
VCTE						
CAP, dB/ms	211 (SD: 32)*	240 (SD: 39)*	232 (SD: 35)	262 (SD: 42)*	288 (SD: 39)*	**<0.001**
LS, kPa^ϯ^	4.3 (3.7-5.0)	4.3 (3.8-4.9)	4.7 (4.0-5.6)	4.5 (3.9-5.0)	5.3 (4.4-6.5)*	**<0.001**
**Bioelectric impedance analysis**						
%Fat	28.2 (SD: 4.4)*	34.7 (SD: 2.6)*	36.7 (SD: 2.9)	39.9 (SD: 3.2)*	42.1 (SD: 4.6)*	**<0.001**
Visceral Fat Rating	4.3 (SD: 1.6)*	6.8 (SD: 1.1)	7.1 (SD: 0.9)	8.5 (SD: 1.4)*	9.8 (SD: 1.8)*	**<0.001**

Data were presented as mean (standard deviation, SD) or median (25^th^–75th percentile).

Binary outcomes were tested using the Cochran–Armitage trend test; continuous outcomes with F-tests for linear trends. Pairwise comparisons were performed using the Holm–Bonferroni correction, with *p < 0.05 considered significant (reference group are the individuals with BMI ≥ 25 kg/m^2^ without confirmed excess adiposity).

ϯLog-transformed before analysis.

eGFR, estimated glomerular filtration rate; FG, fasting glucose; 2hrG, 2-hour post-load glucose; HbA1c, glycated haemoglobin; HOMA-IR, homeostatic model assessment of insulin resistance; SBP, systolic blood pressure; DBP, diastolic blood pressure; HDL-C, high density lipoprotein cholesterol; LDL-C, low density lipoprotein cholesterol; TG, triglycerides; VCTE; vibration controlled transient elastography; CAP, controlled attenuation parameters; LS, liver stiffness.

In the sensitivity analysis, we incorporated percentage of body fat for confirmation of excess adiposity as suggested by the Lancet Commission. Further inclusion of this criterion placed slightly more people in the clinical/preclinical obesity category (S3 Table). Nonetheless, consistent with the main analysis, more men were reclassified as non-obese than women, and more so in the younger subgroup than older subgroup.

### Longitudinal analysis of implications of Lancet Commission definition of obesity on adverse outcomes: results from CRISPS

The baseline characteristics of all 2,900 participants in the CRISPS cohort are summarised in Table 4. 36.2% had BMI ≥25 kg/m^2^ and 22.3% had confirmed excess adiposity defined by elevated WC. Other components of metabolic syndrome were common—hypertension in 18.1%, diabetes in 9.7% and dyslipidaemia in 64.2%. According to the Lancet Commission definition of obesity, of the 1,047 CRISPS participants with BMI ≥25 kg/m^2^, 400 of them would be reclassified as non-obese in view of their normal WC, resulting in a reduction in obesity prevalence to 22.3% (19.7% in men and 24.8% in women).

**Table 4:** Baseline characteristics of CRISPS cohort (n = 2,900).

	All	Men	Women
Number	2,900	1,412	1,488
Age, years	46 (SD: 13)		
<55	2,118 (73.0%)	998 (70.7%)	1,120 (75.3%)
≥55	782 (27.0%)	414 (29.3%)	368 (24.7%)
Smoking status			
Non-smoker	2,160 (74.5%)	739 (52.3%)	1,421 (95.5%)
Ex-smoker	185 (6.4%)	175 (12.4%)	10 (0.7%)
Current smoker	555 (19.1%)	498 (35.3%)	57 (3.8%)
Current alcohol consumption	533 (18.4%)	448 (31.7%)	85 (5.7%)
Regular exercise	1,203 (41.5%)	627 (44.4%)	576 (38.7%)
Education attainment			
No schooling/primary	983 (33.9%)	385 (27.3%)	598 (40.2%)
Secondary	1,516 (52.3%)	777 (55.0%)	739 (49.7%)
Tertiary or above	401 (13.8%)	250 (17.7%)	151 (10.1%)
**Anthropometric parameters**			
BMI^*^, kg/m^2^			
<18.5	116 (4.0%)	48 (3.4%)	68 (4.6%)
18.5–22.9	1,036 (35.8%)	445 (31.5%)	591 (39.8%)
23.0–24.9	697 (24.1%)	376 (26.6%)	321 (21.6%)
25.0–29.9	866 (29.9%)	467 (33.1%)	399 (26.9%)
≥30.0	181 (6.3%)	76 (5.4%)	105 (7.1%)
Elevated waist circumference^ϯ^ (≥90 cm for men; ≥80 cm for women)	736 (25.4%)	306 (21.7%)	430 (28.9%)
Body weight category^*^			
Normal/underweight	1,152 (39.8%)	493 (34.9%)	659 (44.4%)
Overweight	697 (24.1%)	376 (26.6%)	321 (21.6%)
BMI ≥ 25 kg/m^*2*^ without confirmed excess adiposity	400 (13.8%)	264 (18.7%)	136 (9.2%)
Preclinical obesity	346 (11.9%)	153 (10.8%)	193 (13.0%)
Clinical obesity	301 (10.4%)	126 (8.9%)	175 (11.8%)
**Comorbidities**			
Hypertension	525 (18.1%)	260 (18.4%)	265 (17.8%)
Diabetes	280 (9.7%)	137 (9.7%)	143 (9.6%)
Dyslipidaemia	1863 (64.2%)	899 (63.7%)	964 (64.8%)
The cluster of hyperglycaemia, hypertriglyceridaemia and HDL-cholesterol	242 (8.3%)	120 (8.5%)	122 (8.5%)
History of cardiovascular disease	80 (2.8%)	36 (2.5%)	44 (3.0%)
History of cancer	15 (0.5%)	3 (0.2%)	12 (0.8%)

Data were presented as number (%) or mean (standard deviation, SD).

*Four missing data on body weight.

ϯTwo missing data on waist circumference.

**Fig 1:**
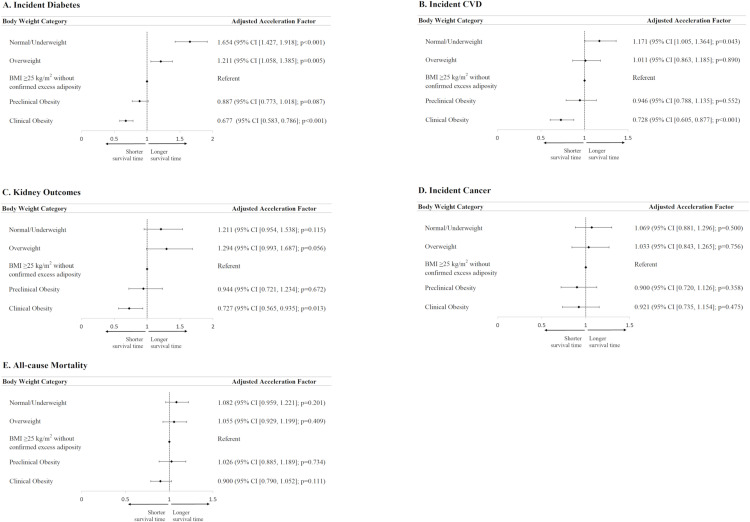
Accelerated failure time (AFT) models to examine the risk of incident cardiovascular-kidney-metabolic outcomes as represented by acceleration factor. Adjusted acceleration factors were estimated from the accelerated failure time (AFT) models and were presented with 95% confidence intervals (CI) and corresponding *p*‑values. *P*‑values were obtained from the Chi‑squared test statistic. All AFT models were fitted using a Weibull distribution, except for cardiovascular and kidney outcomes, which were modelled using a log-normal distribution. Distribution selection was guided by the Akaike Information Criterion (AIC), with lower AIC values indicating superior fit. Covariates in the AFT models included sex, age (modelled with restricted cubic splines), smoking status, current alcohol consumption, regular exercise, and educational attainment.

S4 Table summarises the incidence of cardiovascular-kidney-metabolic outcomes in CRISPS cohort. Upon a median follow-up of over 20 years, 418 participants (19.5%) developed incident diabetes (incidence of 11.2 per 1,000 person-years), 768 participants (27.4%) developed incident cardiovascular diseases (incidence of 11.1 per 1,000 person-years), 113 participants (3.9%) developed adverse kidney outcomes (incidence of 1.5 per 1,000 person-years), 477 participants (17.4%) developed incident cancer (incidence of 8.1 per 1,000 person-years), and 672 participants (23.2%) died during the follow-up period (incidence of 18.0 per 1,000 person-years). Across the range of cardiovascular-kidney-metabolic outcomes, we observed a graded increase in the risk of each of the above outcomes (Figs 1 and S3) from normal/underweight, overweight, BMI ≥25 kg/m^2^ without confirmed excess adiposity, preclinical obesity to clinical obesity. In particular, people who belonged to the category of clinical obesity consistently had the highest risk of adverse outcomes in the AFT model adjusting for sex, age, smoking, alcohol use, exercise and educational level (Fig 1), whereas the group of ‘BMI ≥25kg/m^2^ without confirmed excess adiposity’ consistently had intermediate risk across the five body weight categories.

In the longitudinal cohort of CRISPS, sensitivity analysis to account for possibility of out-migration of the CRISPS participant showed that 514 of the 2,900 individuals enrolled into the CRISPS cohort were identified as not having active follow-up or clinical assessments within the Hospital Authority hospitals. Results from the analyses after excluding these individuals remained consistent with the main analyses (S5 Table).

## Discussion

Using two representative community-based cohorts, we demonstrated that the application of the new definition of clinical/pre-clinical obesity proposed by the Lancet Commission led to reclassification of a small but non-negligible proportion of people who would otherwise have been diagnosed to have obesity based on BMI cut-off alone. Our cross-sectional analysis using N-CRISPS revealed that this group of people being reclassified as non-obese under the Lancet Commission definition was in the intermediate between overweight and pre-clinical obesity, indicated by their degree of insulin resistance and visceral adiposity. Our longitudinal study using CRISPS showed that clinical obesity presents distinctly most adverse clinical risk profile in various cardiovascular-kidney-metabolic outcomes, supporting the benefit of the new classification in directing healthcare resources to target interventions towards this high-risk group of clinical obesity. Nonetheless, in line with the cross-sectional study, people reclassified as non-obese still had more adverse long-term health impacts than those with normal body weight. From the perspective of public health, BMI still serves as an important index to alert the general public to the need to keep themselves at the optimal BMI for their health.

Shortly after the proposal of the new definitions by the Lancet Commission, two studies have evaluated the impact of such new definition on the reclassification of people with obesity diagnosed by high BMI alone and showed different results. Aryee and colleagues studied participants <60 years old in the 2017−2018 National Health and Nutrition Examination Survey (NHANES) as they had DXA data. They reported that 98.4% of people diagnosed to have obesity based on BMI alone were confirmed to have excess adiposity on DXA [15]. Hence, the authors concluded that there was limited utility in confirming excess adiposity among adults in the United States (US) with elevated BMI. On the contrary, the study in United Arab Emirates (UAE) showed that applying the Lancet Commission definition of clinical obesity substantially decreased the prevalence of obesity, potentially leading to a substantial proportion of people missing treatment opportunities [19]. These conflicting findings could be partly explained by the different distribution of ethnicities between the two studies. Although the mean BMI of the cohort in the US study was not reported [15], a much higher rate of severe and extreme obesity defined by BMI [20] in the US could mean limited role of confirming excess adiposity [4]. Furthermore, the UAE study relied heavily on ICD-10 diagnostic codes to identify obesity-related organ dysfunction and might underestimate the prevalence of clinical obesity. Our cross-sectional cohort of N-CRISPS offered novel insights by using a representative community-based cohort derived from validated recruitment method covering a wide age range. Our systematic assessment of a range of cardiometabolic risk factors and availability of body composition by bioelectrical impedance analysis and VCTE led to more accurate classification of clinical obesity. Using validated cut-off points of measurements of central obesity, we found that a non-negligible proportion of people with BMI ≥25 kg/m^2^ were reclassified as non-obese based on the Lancet Commission definition. Although they would not be classified as having clinical/pre-clinical obesity under this new framework, they had a metabolic profile that was intermediate between clinical/pre-clinical obesity and overweight. Their intermediate degree of insulin resistance and proportion of visceral adiposity means that they are likely at an increased risk of adverse health outcomes related to excess adiposity, and still requires attention to mitigate these risks by interventions such as lifestyle measures.

In both CRISPS and N-CRISPS, dyslipidaemia was prevalent—64% and 56%, respectively (Tables 3 and S6). The prevalence of dyslipidaemia increases with increasing body weight categories, mainly driven by the higher triglyceride levels and lower HDL-C levels in relation to the higher degree of insulin resistance with increasing body weight categories. On the other hand, LDL-C levels remain largely similar across the five body weight categories. Studies have suggested that obesity-associated inflammation causes LDL to bind preferentially to collagen in vascular vessel walls over LDL receptors [21], such that LDL-C measurement might not have completely captured how obesity is proatherogenic and contributes to higher cardiovascular risks.

Our study possessed the unique strength of a 20-year longitudinal follow-up to elucidate the long-term cardiovascular-kidney-metabolic implications of implementing the Lancet Commission definition of obesity. Our data reiterated the value of high BMI in predicting multiple adverse cardiovascular-kidney-metabolic outcomes [22]. Moreover, we highlighted the additive value of including WC in the definition of obesity in the prediction of incident cardiovascular-kidney-metabolic outcomes [23]. Our results clearly show that the cardiometabolic risk associated with adiposity is a continuum. We observed a trend across various outcomes with a higher risk among people with clinical/preclinical obesity than those with BMI ≥25 kg/m^2^ but with normal WC. Notably, a crucial piece of evidence from our longitudinal cohort was the highest risk of all cardiovascular-kidney-metabolic outcomes including all-cause mortality among those with clinical obesity, supporting the proposal by the Lancet Commission with real-world evidence that identifying people with clinical obesity can help prioritise interventions to this high-risk population. Nonetheless, we should not ignore those who were diagnosed to have obesity based on BMI cut-off alone but reclassified as non-obese under the Lancet Commission definition, as these people still had higher risk of adverse outcomes than those with normal BMI. Our cohort further demonstrated that these people had higher risk of incident diabetes than those who were overweight, drawing a line between these body weight categories. Taken together, this group of people who are reclassified as non-obese may still benefit from interventions such as lifestyle modifications to optimise their cardiovascular-kidney-metabolic health. The results also serve as food for thought when implementing the Lancet Commission definition to avoid misunderstanding the complete benignity of this category of BMI ≥25 kg/m^2^ without confirmed excess adiposity.

Our study offered insights into the clinical implications of applying the new definitions of obesity proposed by the Lancet Commission using community-based cohorts in a cross-sectional and longitudinal manner. However, our results should be interpreted bearing certain limitations. Firstly, all participants were Chinese and our findings might not apply to other ethnic groups as the epidemiology of obesity differs [24]. Secondly, although we have adopted an inclusive set of criteria to identify obesity-related organ dysfunction, identification of certain obesity-related organ dysfunction relied largely on ICD-9 CM codes, potentially underestimating prevalence of clinical obesity. This might attenuate the difference in the risk profile between pre-clinical and clinical obesity, but would not alter the conclusion that individuals reclassified as non-obese using the new definition still had an intermediate cardiometabolic risk. Thirdly, in the evaluation of the long-term outcomes in CRISPS cohort, while the statistical models adjusted for some social and environmental determinants such as smoking and drinking, exercise and education, we acknowledged that socioeconomic is a broader term that also includes factors such as household overcrowding, per capita income, access/proximity to hospitals, food safety, and rurality status, among others at the individual, interpersonal, and community levels [25]. Importantly, environmental factors are increasingly recognised to contribute to the development of chronic diseases [26]. Last but not least, body fat measurement was not available in the longitudinal cohort of CRISPS, potentially underdiagnosing obesity based on the Lancet Commission definition. Nonetheless, this should not lead to substantial change in the conclusions because we saw from N-CRISPS that only a minority of the people were reclassified to obesity upon inclusion of body fat measurements (Tables 2 and S3).

In conclusion, the Lancet Commission definition works better than the existing BMI-alone criteria in identifying people with clinical obesity to help prioritise interventions to this high-risk population, since people with clinical obesity identified by this revised definition had the highest risks of cardiovascular-kidney-metabolic outcomes including all-cause mortality. On the other hand, adoption of the Lancet Commission definition may have limitations in that a small proportion of individuals with BMI of ≥25.0 kg/m^2^ would be reclassified as non-obese, who in turn had intermediate risks of cardiovascular-kidney-metabolic outcomes between those in pre-clinical obesity and overweight categories.

## Supporting information

S1 Table Operational definitions of the obesity-related organ dysfunction. (DOCX)

S2 Table Whole-body DXA measurements of body composition stratified into five categories (among the subgroup of 55–74 years old). (DOCX)

S3 Table Prevalence of obesity in N-CRISPS cohort based on different criteria of obesity (incorporating percentage of body fat). (DOCX)

S4 Table Incidence of adverse cardiovascular-kidney-metabolic outcomes in CRISPS cohort. (DOCX)

S5 Table Incidence of adverse cardiovascular-kidney-metabolic outcomes in CRISPS cohort (sensitivity analysis excluding possibility of out-migration of the CRISPS participants; reference category is “BMI ≥25kg/m^2^ without confirmed excess adiposity”). (DOCX)

S6 Table Lipid-related parameters in CRISPS, classified according to Lancet Commission definition. (DOCX)

S1 Fig Prevalence of obesity in the N-CRISPS cohort, based on the Lancet Commission’s new definition of obesity, stratified by age and sex. (TIF)

S2 Fig Reclassification of men (*n* = 727) and women (*n* = 433) BMI ≥25 kg/m^2^ based on the Lancet Commission definition of obesity, stratified by age (<55 years and ≥55 years), in the NCRISPS cohort. (TIF)

S3 Fig Kaplan–Meier estimates of incident diabetes (Panel A), incident cardiovascular diseases (Panel B), incident kidney outcomes (Panel C), incident cancer (Panel D), and all-cause mortality (Panel E) among various category at baseline. (TIF)

S1 STROBE Checklist STrengthening the Reporting of OBservational studies in Epidemiology (STROBE) Statement—checklist of items that should be included in reports of observational studies, available at https://www.strobe-statement.org/, licensed under CC BY 4.0. (DOCX)

## Abbreviations

AF: Acceleration Factors
AFT: Accelerated Failure Time
AIC: Akaike Information Criterion
BMI: body mass index
CAP: controlled attenuation parameter
CRISPS: Hong Kong Cardiovascular Risk Factor Prevalence Study
DXA: dual energy X-ray absorptiometry
LS: liver stiffness
N-CRISPS: New Hong Kong Cardiovascular Risk Factor Prevalence Study
SD: standard deviation
STROBE: Strengthening the Reporting of Observational Studies in Epidemiology
VCTE: vibration-controlled transient elastography
WC: waist circumference
WHO: World Health Organization
