# Supplementary material for: The impact of the Lancet Commission definition of obesity on its prevalence and implications on long-term cardiovascular-kidney-metabolic outcomes in East Asians: Observational study of two community-based cohorts
Source: PLoS Med. 2026 Feb 9;23(2):e1004749. doi: 10.1371/journal.pmed.1004749 (PMC12904575; doi:10.1371/journal.pmed.1004749)
Supplement: S1 Table — (DOCX) [file pmed.1004749.s001.docx]

**Supplementary Table 1.** Operational definitions of the obesity-related organ dysfunction

| No. | Organ, Tissue, or Body System | Diagnostic Criterion | Operational Definitions |
| --- | --- | --- | --- |
| 1 | CNS | Signs of raised intracranial pressure such as vision loss and/or recurrent headaches | ICD-9 CM 348.2 |
| 2 | Upper airways | Apnoeas/hypopnoeas during sleep due to increased upper airways resistance | Self report, ICD-9 CM 327.23 |
| 3 | Respiratory | Hypoventilation and/or breathlessness and/or wheezing due to reduced lung and/or diaphragmatic compliance | ICD-9 CM 278.03 |
| 4 | Cardiovascular (ventricular) | Reduced Left Ventricular systolic function - Heart Failure with Reduced Ejection Fraction | Self report, ICD-9 CM 428 |
| 5 | Cardiovascular (atrial) | Chronic/recurrent atrial fibrillation | Self report, ECG, ICD-9 CM 427.31 |
| 6 | Cardiovascular (pulmonary) | Pulmonary artery hypertension | ICD-9 CM 416.0, 416.8 and 416.9 |
| 7 | Cardiovascular | Chronic fatigue, lower limb oedema due to impaired diastolic dysfunction– Heart Failure with Preserved Ejection Fraction | Self report, ICD-9 CM 428 |
| 8 | Recurrent DVT and/or pulmonary thromboembolic disease | Recurrent DVT and/or pulmonary thromboembolic disease | Self report, ICD-9 CM 415.1, 451.1, 451.2, 451.8, 451.9, 452, 453 |
| 9 | Cardiovascular (arterial) | Raised arterial blood pressure | Blood pressure ≥140/90mmHg or on anti-hypertensive medications |
| 10 | Metabolism | The cluster of hyperglycaemia, high triglyceride levels, and low HDL cholesterol levels | Triglyceride ≥1.7mmol/L; HDL-cholesterol <1.0 mmol/L in men, <1.3 mmol/L in women; fasting glucose ≥5.6 mmol/L or already taking oral anti-diabetic agents |
| 11 | Liver | NAFLD with hepatic fibrosis | (For CRISPS) ICD-9 CM 571.8-9  (For N-CRISPS) CAP ≥248 & LS ≥5.8 without heavy drinking or hepatitis B/C |
| 12 | Renal | Microalbuminuria with reduced eGFR | History of albuminuria A2/3 with eGFR <60 mL/min |
| 13 | Urinary | Recurrent/chronic urinary incontinence | ICD-9 CM 788.3 |
| 14 | Reproductive (female) | Anovulation, oligo-menorrhea and PCOS | Self report, ICD-9 CM 256.4 |
| 15 | Reproductive (male) | Male hypogonadism | ICD-9 CM 257.0 |
| 16 | Musculoskeletal | Chronic, severe knee or hip pain associated with joint stiffness and reduced range of joint motion | ICD-9 CM 715.15-16; history of hip or knee replacement |
| 17 | Lymphatic | Lower limbs lymphedema causing chronic pain and/or reduced range of motion | ICD-9 CM 457.1 |
| 18 | Limitations of day-to-day activities | Significant, age-adjusted limitations of mobility and/or other basic Activities of Daily Living | Not available |
