# Supplementary material for: The impact of the Lancet Commission definition of obesity on its prevalence and implications on long-term cardiovascular-kidney-metabolic outcomes in East Asians: Observational study of two community-based cohorts
Source: PLoS Med. 2026 Feb 9;23(2):e1004749. doi: 10.1371/journal.pmed.1004749 (PMC12904575; doi:10.1371/journal.pmed.1004749)
Supplement: S2 Table — (DOCX) [file pmed.1004749.s002.docx]

**Supplementary Table 2.** Whole-body DXA measurements of body composition stratified into five categories (among the subgroup of 55 – 74 years old)

|  | **Normal / underweight** | **Overweight** | **BMI≥25 without confirmed excess adiposity** | **Preclinical Obesity** | **Clinical Obesity** | **P for trend** |
| --- | --- | --- | --- | --- | --- | --- |
|  |  |  | ***(Reference group)*** |  |  |  |
| **Men** |  |  |  |  |  |  |
| **Number** | 93 (33.3) | 79 (28.3) | 17 (6.1) | 15 (5.4) | 75 (26.9) | -- |
| Fat Mass, kg | **14.7±3.1*** | **17.9±2.4*** | 20.3±2.2 | **23.4±3.7*** | **23.3±4.3*** | **<0.001** |
| Android/Gynoid Ratio | 1.08±0.16 | 1.19±0.16 | 1.18±0.12 | 1.20±0.12 | 1.27±0.13 | **<0.001** |
| Trunk/Limb Fat Mass Ratio | **1.13±0.18*** | 1.273±0.20 | 1.265±0.18 | 1.30±0.16 | 1.37±0.20 | **<0.001** |
| Fat Mass/Height^2^ (kg/m^2^) | **5.18±0.99*** | **6.43±0.73*** | 7.32±0.71 | **8.44±1.09*** | **8.39±1.41*** | **<0.001** |
| Appen. Lean/Height^2^ (kg/m^2^) | **6.71±0.64*** | **7.40±0.48*** | 7.93±0.49 | 7.92±0.52 | 8.12±0.71 | **<0.001** |
| Est. VAT Mass (g) | **417±132*** | 559±109 | 642±93 | 726±172 | **796±194*** | **<0.001** |
| Est. VAT Area (cm^2^) | **86±27*** | 116±23 | 133±19 | 151±36 | **165±40*** | **<0.001** |
| Est. VAT Volume (cm^3^) | **451±143*** | 604±118 | 694±100 | 785±186 | **860±210*** | **<0.001** |
|  |  |  |  |  |  |  |
| **Women** |  |  |  |  |  |  |
| **Number** | 127 (52.7) | 55 (22.8) | 3 (1.2) | 18 (7.5) | 38 (15.8) | -- |
| Fat Mass, kg | **17.7±3.0*** | 22.0±2.7 | 26.7±1.1 | 26.1±2.2 | 27.7±4.1 | **<0.001** |
| Android/Gynoid Ratio | 0.92±0.15 | 0.99±0.11 | 0.92±0.08 | 1.04±0.09 | 1.05±0.12 | **<0.001** |
| Trunk/Limb Fat Mass Ratio | 1.00±0.21 | 1.08±0.18 | 0.91±0.09 | 1.09±0.15 | 1.13±0.24 | **<0.001** |
| Fat Mass/Height^2^ (kg/m^2^) | **7.28±1.17*** | **9.05±0.93*** | 11.7±0.3 | 10.8±0.89 | 11.8±1.68 | **<0.001** |
| Appen. Lean/Height^2^ (kg/m^2^) | 5.40±0.45 | 5.85±0.48 | 5.51±0.35 | 6.24±0.51 | **6.54±0.59*** | **<0.001** |
| Est. VAT Mass (g) | 405±142 | 536±169 | 554±159 | 681±161 | 733±209 | **<0.001** |
| Est. VAT Area (cm^2^) | 84±29 | 111±35 | 115±33 | 141±33 | 152±43 | **<0.001** |
| Est. VAT Volume (cm^3^) | 438±154 | 580±182 | 599±172 | 737±174 | 792±226 | **<0.001** |

Est. VAT, Estimated Visceral Adipose Tissue.

Data were presented as mean±standard deviation

Pairwise comparisons were performed using the Bonferroni Correction, with *p<0.05 considered significant

The reference group are individuals with BMI ≥ 25 kg/m^2^ without confirmed excess adiposity
