# Supplementary material for: The impact of the Lancet Commission definition of obesity on its prevalence and implications on long-term cardiovascular-kidney-metabolic outcomes in East Asians: Observational study of two community-based cohorts
Source: PLoS Med. 2026 Feb 9;23(2):e1004749. doi: 10.1371/journal.pmed.1004749 (PMC12904575; doi:10.1371/journal.pmed.1004749)
Supplement: S3 Table — (DOCX) [file pmed.1004749.s003.docx]

**Supplementary Table 3** Prevalence of obesity in N-CRISPS cohort based on different criteria of obesity (incorporating percentage of body fat)

|  | **Number** | **Obesity defined by BMI≥25 kg/m^2^ alone** | **Obesity defined by BMI and confirmed excess adiposity (by either elevated waist circumference or percentage of body fat)** | | |
| --- | --- | --- | --- | --- | --- |
|  |  |  | **Preclinical Obesity** | **Clinical Obesity** | **Total** |
| Men |  |  |  |  |  |
| 25-54y | 1005 | 457 (45.5%) | 143 (14.2%) | 215 (21.4%) | 358 (35.6%) |
| 55-74y | 630 | 270 (42.9%) | 39 (6.2%) | 206 (32.7%) | 245 (38.9%) |
| Total | 1635 | 727 (44.5%) | 182 (11.1%) | 421 (25.7%) | 603 (36.8%) |
|  |  |  |  |  |  |
| Women |  |  |  |  |  |
| 25-54y* | 1018 | 248 (24.4%) | 131 (12.9%) | 107 (10.5%) | 238 (23.4%) |
| 55-74y | 605 | 185 (30.6%) | 60 (9.9%) | 125 (20.7%) | 185 (30.6%) |
| Total | 1623 | 433 (26.7%) | 191 (11.8%) | 232 (14.3%) | 423 (26.1%) |

*Fat mass data for one participant was unavailable due to the presence of pacemaker – her BMI was ≥25 kg/m^2^, but her WC was not elevated such that obesity defined by BMI in combination with either elevated waist circumference or body fat mass could not be determined
