# Supplementary material for: The impact of the Lancet Commission definition of obesity on its prevalence and implications on long-term cardiovascular-kidney-metabolic outcomes in East Asians: Observational study of two community-based cohorts
Source: PLoS Med. 2026 Feb 9;23(2):e1004749. doi: 10.1371/journal.pmed.1004749 (PMC12904575; doi:10.1371/journal.pmed.1004749)
Supplement: S4 Table — (DOCX) [file pmed.1004749.s004.docx]

**Supplementary Table 4.** Incidence of adverse cardiovascular-kidney-metabolic outcomes in CRISPS cohort

|  | **Diabetes** | **Cardiovascular diseases** | **Cancer** | **Kidney outcomes** | **All-cause Mortality** |
| --- | --- | --- | --- | --- | --- |
| **Number** | 2139 | 2802 | 2874 | 2894 | 2900 |
| **Censored date** | C2/C3/C4/C5 | 2024-12-31 | 2024-12-31 | 2024-12-31 | 2024-12-31 |
| **Median follow-up, year** | 21 (13-23) | 29 (22-29.5) | 23 (22.6-24) | 29 (28-30) | 29 (28-29.6) |
| **Number of events** | 418 | 768 | 477 | 113 | 672 |
| **Cumulative incidence** | 19.5% | 27.4% | 17.4% | 3.9% | 23.2% |
| **Incident rate, per 1000 years** | 11.19 | 11.09 | 6.47 | 1.47 | 18.04 |

For analysis of the specified outcome, participants who had the disease at baseline and a follow-up period exceeding one year were included.
