# Supplementary material for: The impact of the Lancet Commission definition of obesity on its prevalence and implications on long-term cardiovascular-kidney-metabolic outcomes in East Asians: Observational study of two community-based cohorts
Source: PLoS Med. 2026 Feb 9;23(2):e1004749. doi: 10.1371/journal.pmed.1004749 (PMC12904575; doi:10.1371/journal.pmed.1004749)
Supplement: S5 Table — (DOCX) [file pmed.1004749.s005.docx]

**Supplementary Table 5.** Incidence of adverse cardiovascular-kidney-metabolic outcomes in CRISPS cohort (sensitivity analysis excluding possibility of out-migration of the CRISPS participants; reference category is ‘BMI≥25kg/m^2^ without confirmed excess adiposity’)

| **Body weight category** | **Cardiovascular diseases** | **Kidney outcomes** | **Cancer** | **All-cause mortality** |
| --- | --- | --- | --- | --- |
| **Normal/ Underweight** | 1.147 (0.987-1.333)  p=0.073 | 1.168 (0.937-1.456)  p=0.168 | 1.056 (0.905-1.231)  p=0.492 | 1.059 (0.944-1.188)  p=0.326 |
| **Overweight** | 0.974 (0.833-1.139)  p=0.741 | 1.261 (0.984-1.615)  p=0.0667 | 1.020 (0.867-1.198)  p=0.815 | 0.997 (0.883-1.127)  p=0.968 |
| **Pre-clinical obesity** | 0.946 (0.791-1.132)  p=0.5451 | 0.936 (0.731-1.199)  p=0602 | 0.938 (0.784-1.123)  p=0.488 | 1.004 (0.873-1.155)  p=0.954 |
| **Clinical obesity** | 0.735 (0.614-0.880)  **p<0.001** | 0.758 (0.601-0.956)  **p=0.019** | 0.943 (0.788-1.128)  p=0.521 | 0.875 (0.773-0.990)  **p=0.034** |

Data presented as acceleration factors and their confidence intervals with p-values.

Association between body weight categories and incident diabetes would not require separate sensitivity analysis as since we included participants who had attended at least one follow-up visit after baseline in the main analysis.
