# Supplementary material for: The impact of the Lancet Commission definition of obesity on its prevalence and implications on long-term cardiovascular-kidney-metabolic outcomes in East Asians: Observational study of two community-based cohorts
Source: PLoS Med. 2026 Feb 9;23(2):e1004749. doi: 10.1371/journal.pmed.1004749 (PMC12904575; doi:10.1371/journal.pmed.1004749)
Supplement: S6 Table — (DOCX) [file pmed.1004749.s006.docx]

**Supplementary Table 6.** Lipid-related parameters in CRISPS, classified according to Lancet Commission definition

| **Men (N=1412)** | | | | | | | |
| --- | --- | --- | --- | --- | --- | --- | --- |
|  | **Non-obese group** | | |  | **Obesity group** | | **P for trend** |
|  | **Normal / underweight**  **BMI <23 kg/m^2^** | **Overweight**  **BMI 23-24.9 kg/m^2^** | **BMI ≥25 kg/m^2^ without confirmed adiposity**  ***(Reference group)*** |  | **Preclinical Obesity** | **Clinical Obesity** |  |
| **Number** | 493 | 376 | 264 |  | 153 | 126 | **--** |
| Dyslipidaemia | 223 (45.3%)* | 234 (62.4%)* | 202 (76.5%) |  | 123 (80.4%) | 117 (92.9%)* | <**0.001** |
| HDL-C, mmol/L | 1.29 (SD: 0.32)* | 1.17 (SD: 0.28) | 1.07 (SD: 0.25) |  | 1.01 (SD: 0.22)* | 0.98 (SD: 0.24)* | **<0.001** |
| LDL-C, mmol/L | 3.2 (SD: 0.8)* | 3.4 (SD: 0.9)* | 3.5 (SD: 0.9) |  | 3.4 (SD: 0.7) | 3.5 (SD: 0.8) | **<0.001** |
| TG, mmol/Lϯ | 0.9 (0.7-1.2)* | 1.2 (0.8-1.6)* | 1.3 (0.9-1.9) |  | 1.4 (1.1-1.7) | 1.9 (1.4-2.6)* | **<0.001** |
|  |  |  |  |  |  |  |  |
| **Women (N=1488)** | | | | | | | |
|  | **Non-obese group** | | |  | **Obesity group** | | **P for trend** |
|  | **Normal / underweight**  **BMI <23 kg/m^2^** | **Overweight**  **BMI 23-24.9 kg/m^2^** | **BMI ≥25 kg/m^2^ without confirmed adiposity**  ***(Reference group)*** |  | **Preclinical Obesity** | **Clinical Obesity** |  |
| **Number** | 659 | 321 | 136 |  | 193 | 175 | **--** |
| Dyslipidaemia | 331 (50.4%)* | 211 (65.7%) | 104 (76.5%) |  | 157 (81.8%) | 159 (90.9%)* | <**0.001** |
| HDL-C, mmol/L | 1.44 (SD: 0.31)* | 1.34 (SD: 0.33) | 1.30 (SD: 0.27) |  | 1.26 (SD: 0.28) | 1.12 (SD: 0.27)* | **<0.001** |
| LDL-C, mmol/L | 3.0 (SD: 0.9)* | 3.2 (SD: 1.0) | 3.3 (SD: 0.9) |  | 3.4 (SD: 0.8) | 3.6 (SD: 0.9)* | **<0.001** |
| TG, mmol/Lϯ | 0.8 (0.6-1.0*) | 0.9 (0.6-1.3)* | 1.0 (0.7-1.2) |  | 1.1 (0.8-1.4)* | 1.7 (1.1-2.3)* | **<0.001** |

Data were presented as mean (standard deviation, SD) or median (25^th^ – 75^th^ percentile). ϯLog-transformed before analysis.

Pairwise comparisons were performed using the Holm-Bonferroni correction, with *p<0.05 considered significant (reference group are the individuals with BMI ≥ 25kg/m^2^ without confirmed excess adiposity).

HDL-C, high density lipoprotein cholesterol; LDL-C, low density lipoprotein cholesterol; TG, triglycerides.
